# Revisiting the role of pregnancy zone protein (PZP) as a cancer biomarker in the immunotherapy era

**DOI:** 10.1186/s12967-024-05321-5

**Published:** 2024-05-26

**Authors:** Jie Huang, Ying Xu, Yidan Chen, Juan Shen, Yao Qiu, Xin Li, Xueqin Chen, Shenglin Ma

**Affiliations:** 1 Department of Oncology, Hangzhou Cancer Hospital, No.34 Yanguan Lane, Shangcheng District, Hangzhou, 310002 China; 2 The Forth Clinical Medical College, Zhejiang Chinese Medical University, Hangzhou, 310006 China; 3 Cancer Research Institution, Hangzhou Cancer Hospital, Hangzhou, 310002 China; 4 Affiliated Hangzhou First People’s Hospital, School of Medicine, Westlake University, Hangzhou, 310006 China

## To the Editor,

Immunotherapy with PDL1/PD1 or CTLA4 based immune checkpoint inhibitors has greatly improved survival in various cancers, however, the efficacy is limited and cancer as an aggressive disease still faces many unmet needs. Pregnancy related proteins have been associated with carcinogenesis, in which alpha fetoprotein (AFP) and carcinoembryonic antigen (CEA) have been widely used as tumor markers in cancer diagnosis, prognosis and therapy response evaluation. Pregnancy zone protein (PZP), as another pregnancy related protein, is abundantly secreted in the plasma by placenta during pregnancy [1]. was initially evaluated as a potential tumor marker but have been demonstrated to be unsuitable due to no apparent associations between PZP plasma levels and either tumor burden or treatment response [2, 3]. However, we also noticed that recent publications demonstrated a clear role of PZP for screening lung adenocarcinoma in type 2 diabetes mellitus patients [4], which hints the possible usage of PZP as a biomarker in specific circumstances. And notably it is worth emphasizing that a comprehensive evaluation of PZP in various cancers especially associated tumor immune microenvironment and immunotherapy response are still needed in the current tumor immunotherapy era since PZP, classically recognized as a pan-protease inhibitor, mediates immune tolerance during pregnancy [5], implicating its possible role in regulation of tumor immune microenvironment. Hence, we performed a pan-cancer analysis of PZP to reveal its expression levels and prognosis indications in various cancer types and its links with cancer hallmarks especially the associations with tumor immune microenvironment and immunotherapy responses.

## Findings

### The distinct expression and prognosis indications of PZP in various cancers

**Fig. 1:**
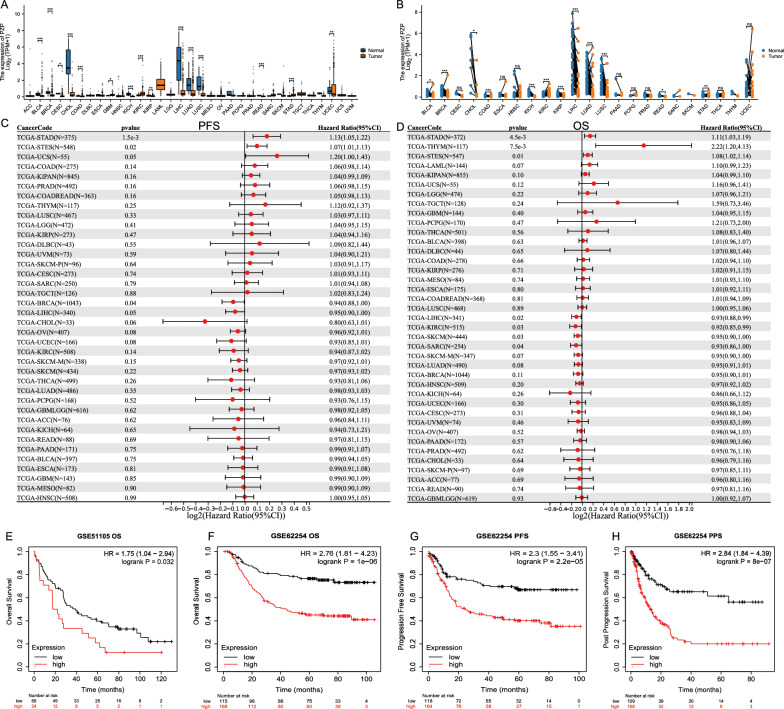
PZP expression and its prognostic relevance across different cancer types in pan-cancer analysis. **A**, **B** Expression variations of PZP between cancer and peritumoral tissues (Wilcoxon rank sum test) (**A**) and paired cancer and peritumoral tissues (Wilcoxon signed rank test) (**B**) in various cancer cohorts from the TCGA database. Symbols “*”, “**”, and “***” denote statistical significance with p < 0.05, p < 0.01, and p < 0.001, respectively. **C**, **D** Forest plot of univariate Cox regression analysis illustrating the HRs of PZP in pan-cancer for PFS **(C)** and OS **(D)**. **E–H** Survival plots of Kaplan–Meier log-rank analysis of OS between PZP low and PZP high expression stomach adenocarcinoma patients grouped by best cut-off values of PZP in STAD dataset GSE51105 **(E)** and OS **(F)**, PFS **(G)** and PPS **(H)** in STAD dataset GSE62254

Through comparing the expression levels of PZP among cancers and their peritumor normal tissues in The Cancer Genome Atlas (TCGA), we found that PZP expression in most cancer tissues including BLCA, BRCA, CESC, CHOL, COAD, KICH, KIRP, LIHC, LUAD, LUSC, READ, and UCEC were significantly lower than their peritumor normal tissues, while only GBM, KIRC, and STAD cancer types had higher PZP expression levels than their normal tissues (P < 0.05) **(**Fig. 1A**) (**full names and abbreviations of cancer types in TCGA were listed Supplementary Table S1). Comparisons between paired tumor-normal tissues also confirmed decreased PZP expressions in BLCA, BRCA, CHOL, COAD, KICH, KIRP, LIHC, LUAD, LUSC, READ cancer types but increased expressions in KIRC and STAD (P < 0.05) **(**Fig. 1B**)**. Then, we wondered whether PZP might be a risk factor in specific cancer types and the univariate COX regression analyses in different cancers in TCGA revealed that PZP indicated a good progression-free survival (PFS) in BRCA and a good overall survival (OS) in LIHC, KIRC, SKCM, and SARC, but a poor PFS in STAD, STES and a poor OS in STAD, THYM, and STES (P < 0.05) **(**Fig. 1C, D**) **(Supplementary Figs. S1, S2). Notably, PZP was further validated as a risk factor of OS by another two independent STAD datasets including GSE51105 **(**Fig. 1E**)** and GSE62254 **(**Fig. 1F**)**. Additionally, higher PZP expressions also indicated worse PFS **(**Fig. 1G**)** and post-progression survival (PPS) **(**Fig. 1H**)**, as demonstrated by STAD dataset GSE62254.

### PZP linked with tumor immune microenvironment and immunotherapy response

**Fig. 2:**
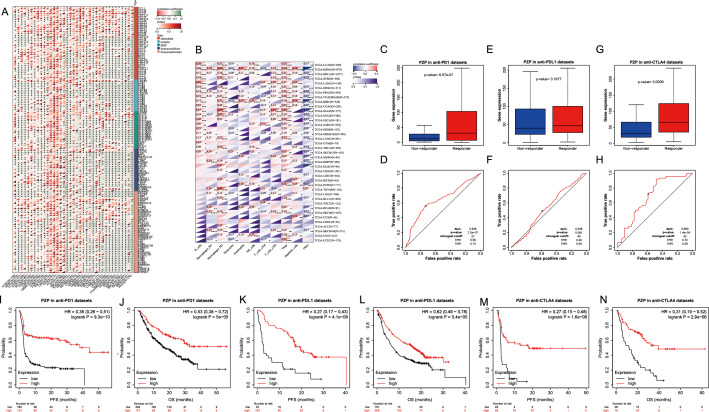
Correlations between PZP expression and immune-related genes, immune microenvironment and immunotherapy response.** A** Heat maps of associations between PZP expression and immune regulator genes including chemokines and chemokine receptors, MHC, immunoinhibitory or immunostimulatory genes in pan-cancer. Symbols “*”, “**”, and “***” denote statistical significance with p < 0.05, p < 0.01, and p < 0.001, respectively (Pearson correlation). **B** Heat maps of associations between PZP expression and infiltrated immune cells analyzed by QUANTISEQ algorithm in pan cancer. Symbols “*”, “**”, and “***” denote statistical significance with p < 0.05, p < 0.01, and p < 0.001, respectively (Pearson correlation). **C**, **E**, **G** Box plots showing the PZP expression levels between responders and non-responders to anti-PD1 **(C)**, anti-PDL1**(E)**, and anti-CTLA4**(G)** immunotherapy datasets. **D**, **F**, **H** ROC curves showing the AUC values of PZP to predict response to anti-PD1 **(D)**, anti-PDL1**(F)**, and anti-CTLA4 **(H)** immunotherapy. **I**–**N** Survival plots of Kaplan–Meier log-rank analysis of PFS and OS between PZP low and PZP high expression patients grouped by best cut-off values in cancer patients receiving PD1 inhibitors** (I**, **J)**, PDL1 inhibitors** (K**, **L)**, CTLA4 inhibitors **(M**, **N)** as analyzed by Kaplan Meier Plotter

Regarding the role of PZP in mediating tumor immune evasion, we checked the correlation of PZP expressions with immune regulator genes. Remarkably, the results demonstrated positive correlations of PZP expressions with most immune checkpoint genes including well-known CD274, CTLA4, TIGIT, LAG3, etc., chemokines, receptors, MHC, and other immune regulators in most cancers including STAD **(**Fig. 2A**)**. When we delve into the detailed immune cell subtypes infiltrated into the tumor microenvironment, we noted that PZP expression was positively correlated with M2 macrophages and Tregs in most cancers **(**Fig. 2B**)**. Notably, when exploring the impact of PZP expression on immunotherapy response using immunotherapy datasets in Kaplan–Meier Plotter, we unexpectedly found that responders to anti-PD1 or anti-CTLA4 immunotherapy had significantly higher PZP expression than non-responders **(**Fig. 2C, G**)** and PZP could predict the response to anti-PD1 and anti-CTLA4 immunotherapy with AUC of 0.646 and 0.693, respectively **(**Fig. 2D, H**)**, and a insignificant trend in anti-PDL1 immunotherapy datasets **(**Fig. 2E, F**)**. Patients with higher PZP expression had obviously better PFS and OS compared to lower ones when treated with either PD1 inhibitors **(**Fig. 2I, J**)**, PDL1 inhibitors **(**Fig. 2K, L**)**, or CTLA4 inhibitors **(**Fig. 2M, N**)**.

## Conclusions

In summary, we performed a comprehensive evaluation of PZP in various cancers, which revealed its underlying role as a prognostic indicator several cancer types including STAD and its links with immune microenvironment. PZP widely regulates immune regulators including immune checkpoint genes, facilitates the immune-tolerant tumor microenvironment, and predicts the immunotherapy response. Thus, PZP may become a new biomarker guiding PD1 or CTLA4 based immunotherapy in cancers.

### Supplementary Information

Supplementary Material 1. Figure S1. Kaplan-Meier survival curves for PFS in cancers stratified by expression of PZP. A-E. Kaplan-Meier survival curves for PFS in STES (A), UCS (B), LIHC (C), STAD (D), BRCA (E) stratified by expression of PZP.

Supplementary Material 2. Figure S2. Kaplan-Meier survival curves for OS in cancers stratified by expression of PZP. A-G. Kaplan-Meier survival curves for OS in THYM (A), STES (B), STAD (C), SKCM (D), SARC (E), LIHC (F) and KIRC (G) stratified by expression of PZP.

Supplementary Material 3. Supplementary Table S1. Full names and abbreviations of enrolled cohorts in the TCGA database.

## Data Availability

The dataset supporting the conclusions of this article is included within the article. The data supporting the findings of this study are deposited in the TCGA and GEO (GSE51105, GSE62254 for STAD prognosis validation), and Kaplan–Meier Plotter for immunotherapy response evaluation (https://kmplot.com/analysis/index.php?p=service&cancer=immunotherapy).

## Abbreviations

PZP: Pregnancy zone protein
PD-1: Programed-cell death protein 1
PD-L1: Programed-cell death-ligand 1
CTLA-4: Cytotoxic T-lymphocyte associated protein 4
AFP: Alpha fetoprotein
CEA: Carcinoembryonic antigen
TCGA: The Cancer Genome Atlas
PFS: Progression-free survival
OS: Overall survival
PPS: Post-progression survival
CD274: Cluster of differentiation 274
TIGIT: T-cell immunoreceptor with Ig and ITIM domains
LAG3: Lymphocyte-activation gene 3
