# Supplementary figures and images for: Revisiting the role of pregnancy zone protein (PZP) as a cancer biomarker in the immunotherapy era
Source: J Transl Med. 2024 May 26;22:500. doi: 10.1186/s12967-024-05321-5 (PMC11128099; doi:10.1186/s12967-024-05321-5)

**A**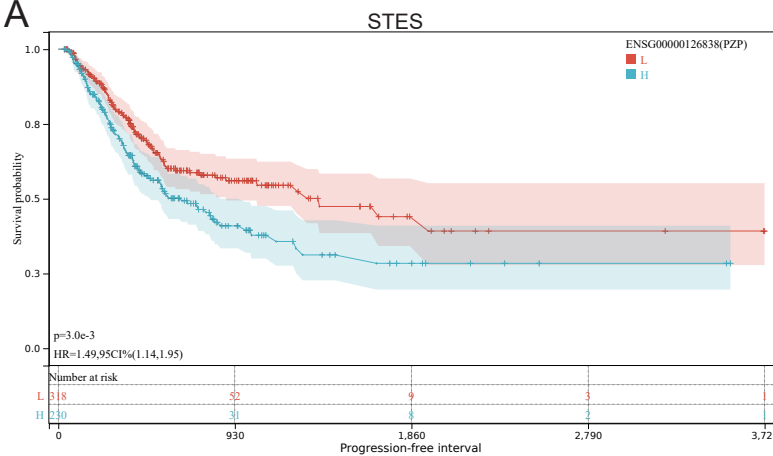**B**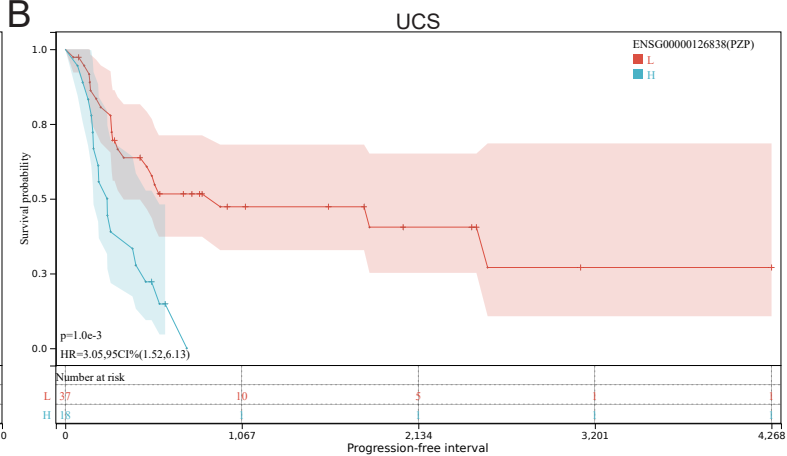**C**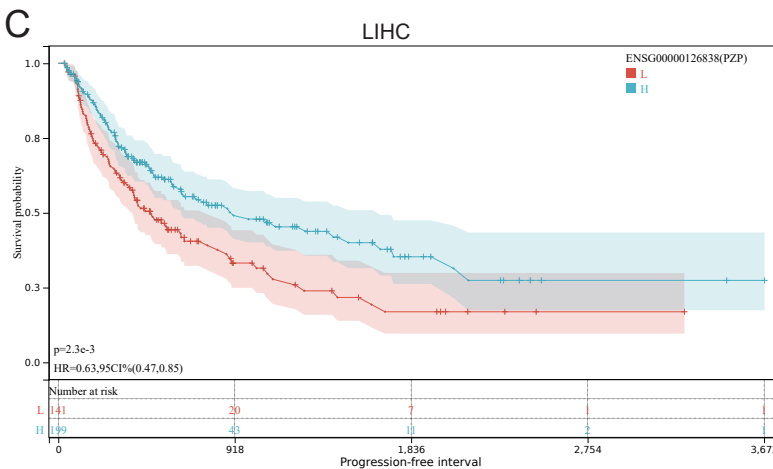**D**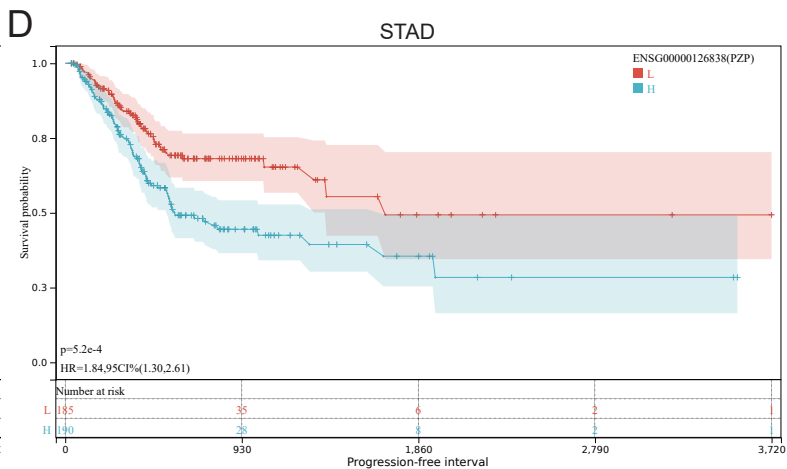**E**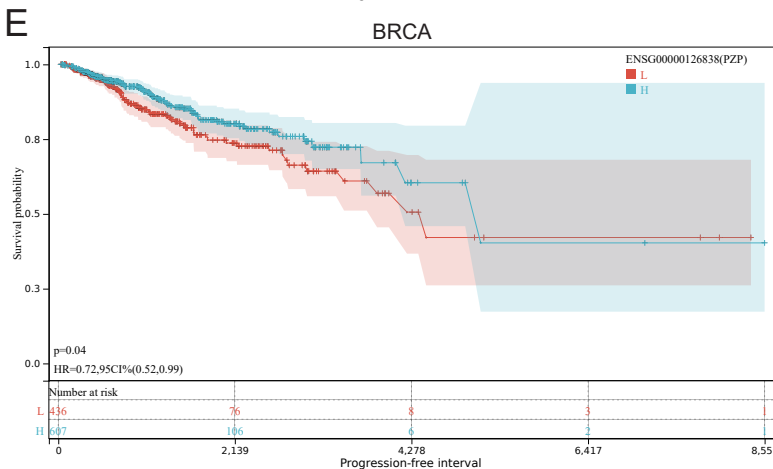

Supplement: Supplementary file 1 — Supplementary Material 1. Figure S1. Kaplan-Meier survival curves for PFS in cancers stratified by expression of PZP. A-E. Kaplan-Meier survival curves for PFS in STES (A), UCS (B), LIHC (C), STAD (D), BRCA (E) stratified by expression of PZP. [file 12967_2024_5321_MOESM1_ESM.pdf]

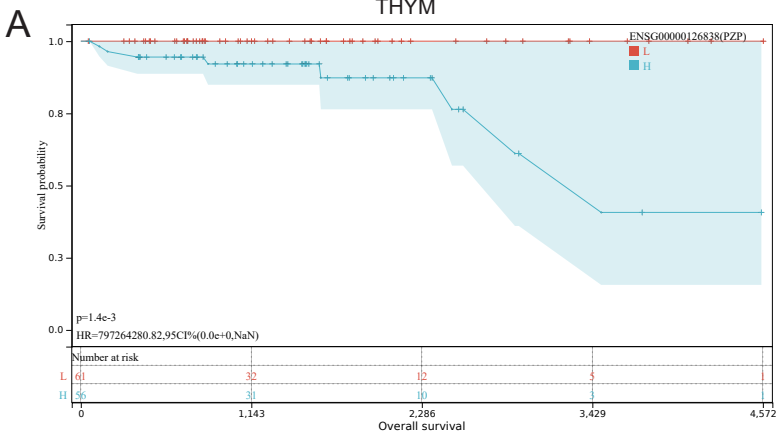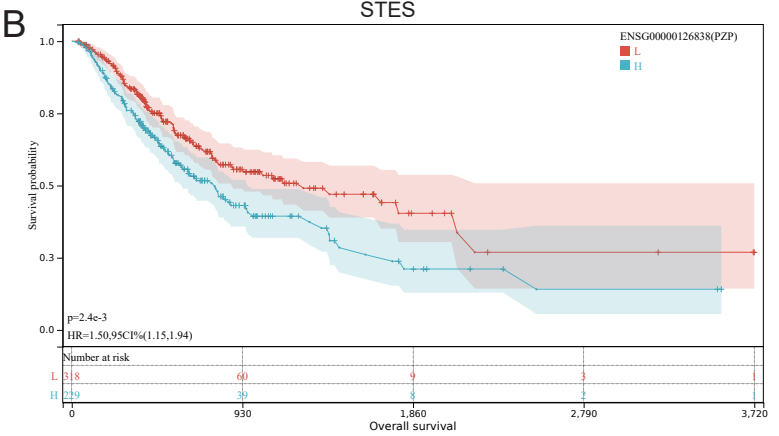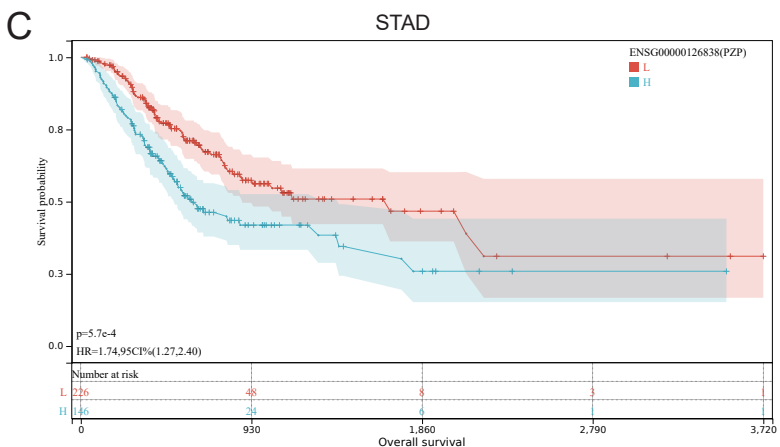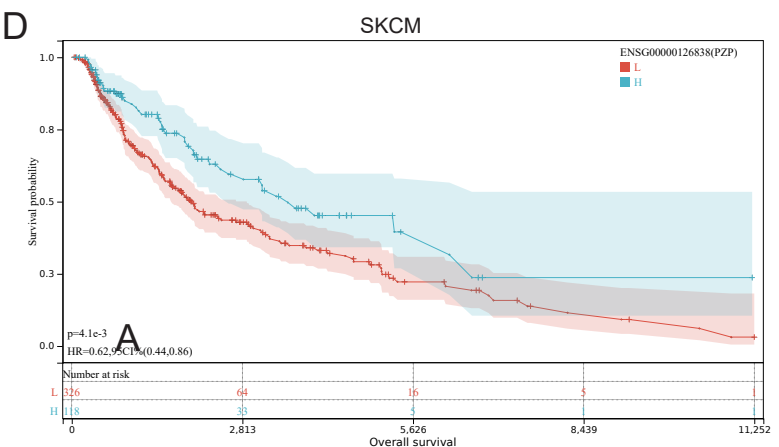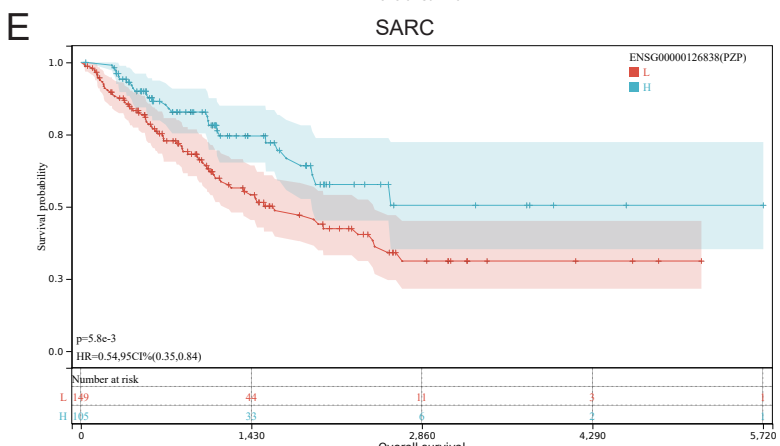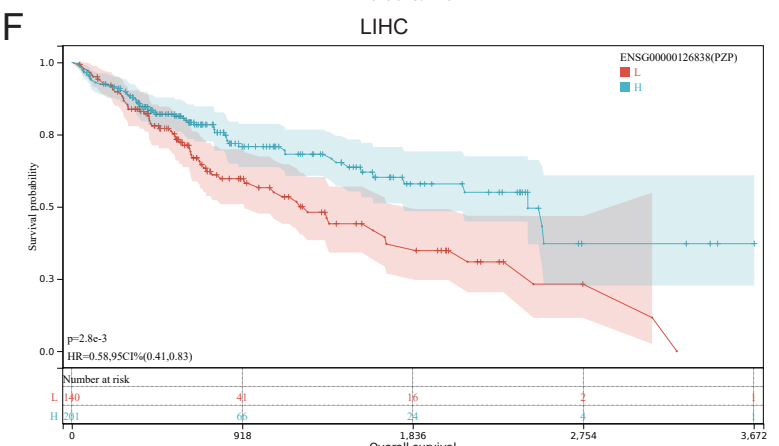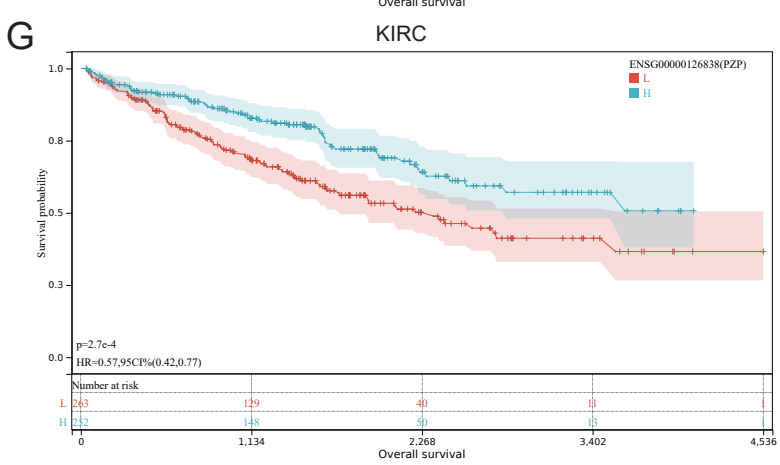

Supplement: Supplementary file 2 — Supplementary Material 2. Figure S2. Kaplan-Meier survival curves for OS in cancers stratified by expression of PZP. A-G. Kaplan-Meier survival curves for OS in THYM (A), STES (B), STAD (C), SKCM (D), SARC (E), LIHC (F) and KIRC (G) stratified by expression of PZP. [file 12967_2024_5321_MOESM2_ESM.pdf]
