# Supplementary material for: Revisiting the role of pregnancy zone protein (PZP) as a cancer biomarker in the immunotherapy era
Source: J Transl Med. 2024 May 26;22:500. doi: 10.1186/s12967-024-05321-5 (PMC11128099; doi:10.1186/s12967-024-05321-5)
Supplement: Supplementary file 3 — Supplementary Material 3. Supplementary Table S1. Full names and abbreviations of enrolled cohorts in the TCGA database. [file 12967_2024_5321_MOESM3_ESM.docx]

| Abbreviations | Name |
| --- | --- |
| ACC | Adrenocortical Carcinoma |
| BLCA | Bladder Urothelial Carcinoma |
| BRCA | Breast Invasive Carcinoma |
| CESC | Cervical Squamous Cell Carcinoma and Endocervical Adenocarcinoma |
| CHOL | Cholangiocarcinoma |
| COAD | Colon Adenocarcinoma |
| DLBC | Lymphoid Neoplasm Diffuse Large B-cell Lymphoma |
| ESCA | Esophageal Carcinoma |
| GBM | Glioblastoma Multiforme |
| GBMLGG | Glioma |
| HNSC | Head and Neck Squamous Cell Carcinoma |
| KICH | Kidney Chromophobe |
| KIRC | Kidney Renal Clear Cell Carcinoma |
| KIRP | Kidney Renal Papillary Cell Carcinoma |
| LAML | Acute Myeloid Leukemia |
| LGG | Brain Lower Grade Glioma |
| LIHC | Liver Hepatocellular Carcinoma |
| LUAD | Lung Adenocarcinoma |
| LUSC | Lung Squamous Cell Carcinoma |
| MESO | Mesothelioma |
| OV | Ovarian Serous Cystadenocarcinoma |
| PAAD | Pancreatic Adenocarcinoma |
| PRAD | Prostate Adenocarcinoma |
| READ | Rectum Adenocarcinoma |
| SARC | Sarcoma |
| SKCM | Skin Cutaneous Melanoma |
| STES | Stomach and Esophageal carcinoma |
| STAD | Stomach Adenocarcinoma |
| TGCT | Testicular Germ Cell Tumors |
| THCA | Thyroid Carcinoma |
| THYM | Thymoma |
| UCEC | Uterine Corpus Endometrial Carcinoma |
| UCS | Uterine Carcinosarcoma |
| UVM | Uveal Melanoma |

Supplementary Table 1: Full names and abbreviations of enrolled cohorts in the TCGA database.
